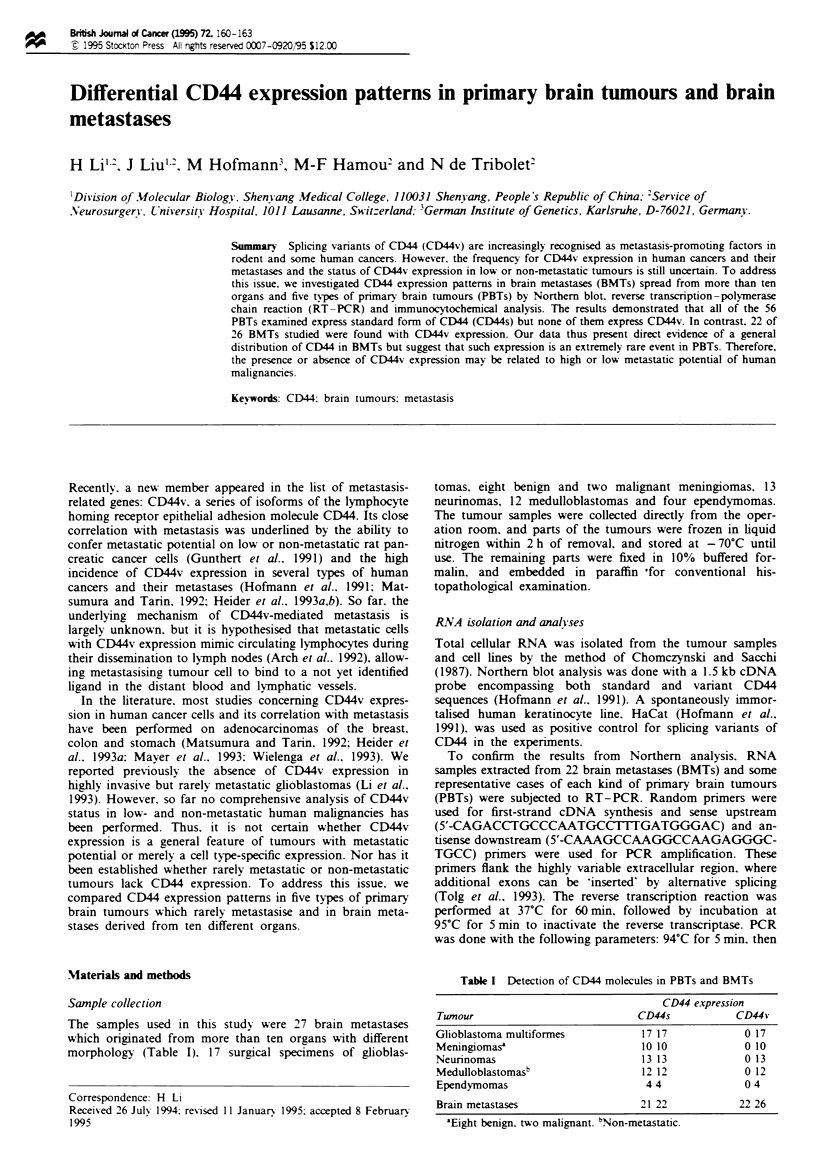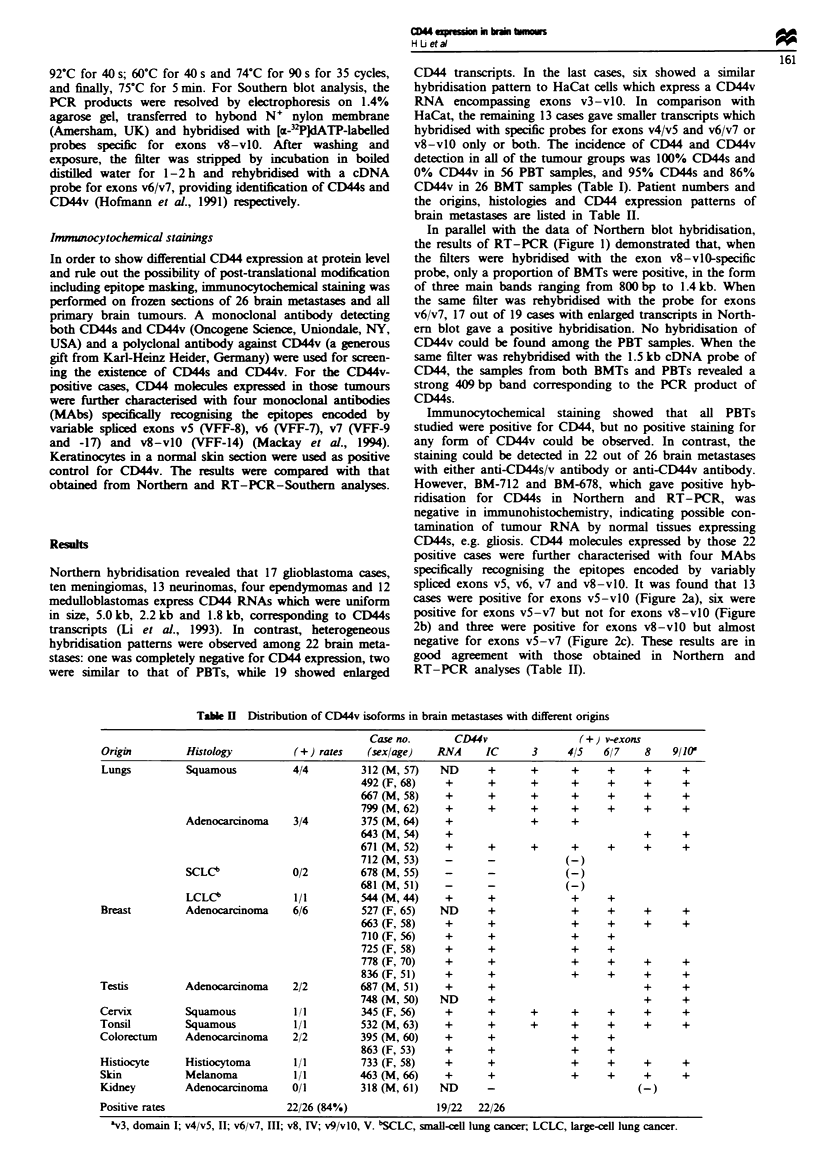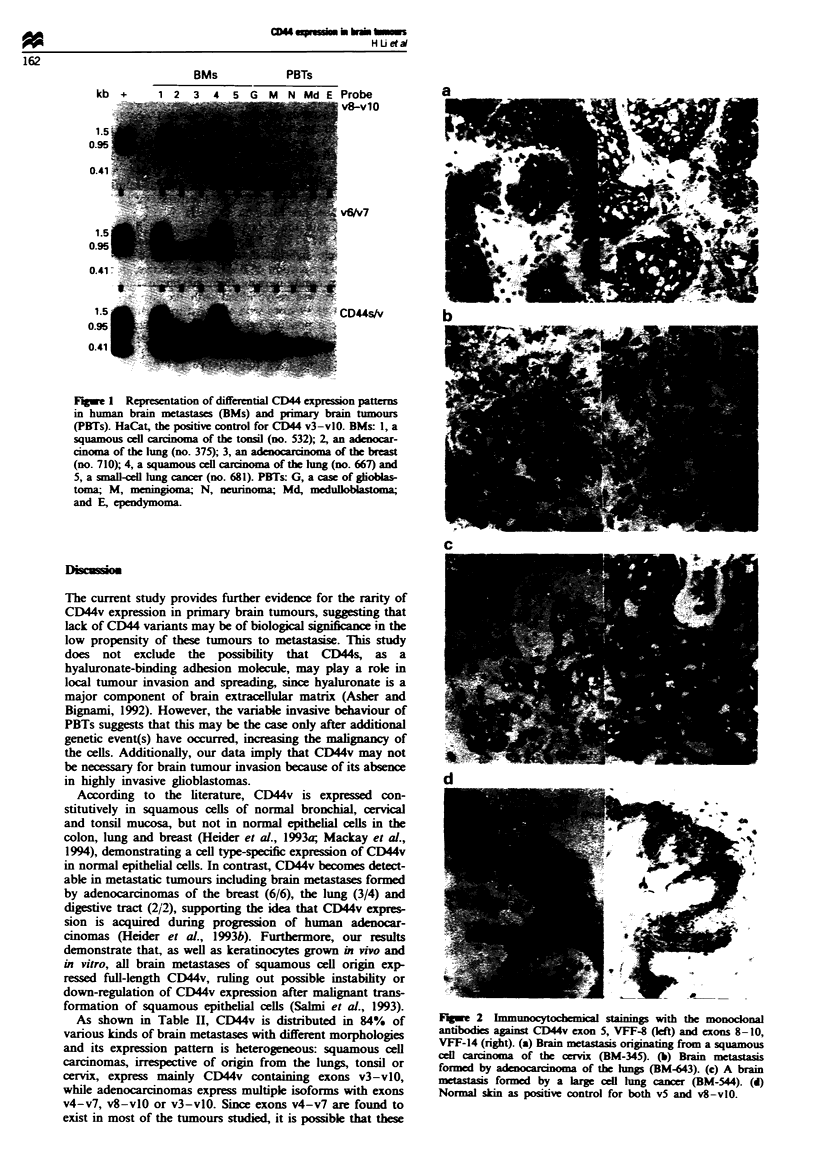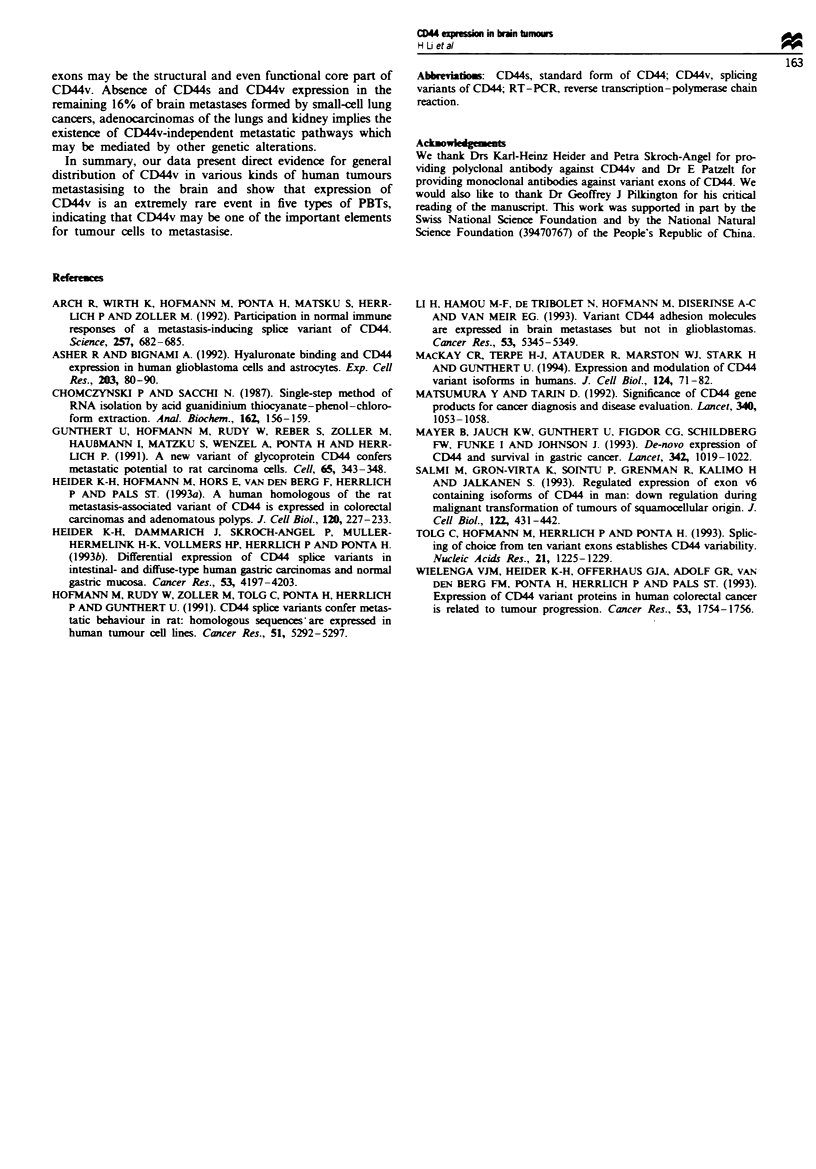# Differential CD44 expression patterns in primary brain tumours and brain metastases.

**DOI:** 10.1038/bjc.1995.294

**Published:** 1995-07

**Authors:** H. Li, J. Liu, M. Hofmann, M. F. Hamou, N. de Tribolet

**Affiliations:** Division of Molecular Biology, Shenyang Medical College, People's Republic of China.

## Abstract

**Images:**


© 1995 Stockton Press All rights reserved 0007-0920/95 $12.00

### Differential CD44 expression patterns in primary brain tumours and brain metastases

H Li[1,2], J Liu[1,2], M Hofmann[3], M-F Hamou[2] and N de Tribolet[2]

[1]*Division of Molecular Biology, Shenyang Medical College, 110031 Shenyang, People's Republic of China;* [2]*Service of Neurosurgery, University Hospital, 1011 Lausanne, Switzerland;* [3]*German Institute of Genetics, Karlsruhe, D-76021, Germany.*

**Summary** Splicing variants of CD44 (CD44v) are increasingly recognised as metastasis-promoting factors in rodent and some human cancers. However, the frequency for CD44v expression in human cancers and their metastases and the status of CD44v expression in low or non-metastatic tumours is still uncertain. To address this issue, we investigated CD44 expression patterns in brain metastases (BMTs) spread from more than ten organs and five types of primary brain tumours (PBTs) by Northern blot, reverse transcription-polymerase chain reaction (RT-PCR) and immunocytochemical analysis. The results demonstrated that all of the 56 PBTs examined express standard form of CD44 (CD44s) but none of them express CD44v. In contrast, 22 of 26 BMTs studied were found with CD44v expression. Our data thus present direct evidence of a general distribution of CD44 in BMTs but suggest that such expression is an extremely rare event in PBTs. Therefore, the presence or absence of CD44v expression may be related to high or low metastatic potential of human malignancies.

**Keywords**: CD44; brain tumours; metastasis

Recently, a new member appeared in the list of metastasis-related genes: CD44v, a series of isoforms of the lymphocyte homing receptor epithelial adhesion molecule CD44. Its close correlation with metastasis was underlined by the ability to confer metastatic potential on low or non-metastatic rat pancreatic cancer cells (Gunthert *et al.*, 1991) and the high incidence of CD44v expression in several types of human cancers and their metastases (Hofmann *et al.*, 1991; Matsumura and Tarin, 1992; Heider *et al.*, 1993*a*,*b*). So far, the underlying mechanism of CD44v-mediated metastasis is largely unknown, but it is hypothesised that metastatic cells with CD44v expression mimic circulating lymphocytes during their dissemination to lymph nodes (Arch *et al.*, 1992), allowing metastasising tumour cell to bind to a not yet identified ligand in the distant blood and lymphatic vessels.

In the literature, most studies concerning CD44v expression in human cancer cells and its correlation with metastasis have been performed on adenocarcinomas of the breast, colon and stomach (Matsumura and Tarin, 1992; Heider *et al.*, 1993*a*; Mayer *et al.*, 1993; Wielenga *et al.*, 1993). We reported previously the absence of CD44v expression in highly invasive but rarely metastatic glioblastomas (Li *et al.*, 1993). However, so far no comprehensive analysis of CD44v status in low- and non-metastatic human malignancies has been performed. Thus, it is not certain whether CD44v expression is a general feature of tumours with metastatic potential or merely a cell type-specific expression. Nor has it been established whether rarely metastatic or non-metastatic tumours lack CD44 expression. To address this issue, we compared CD44 expression patterns in five types of primary brain tumours which rarely metastasise and in brain metastases derived from ten different organs.

### Materials and methods

### Sample collection

The samples used in this study were 27 brain metastases which originated from more than ten organs with different morphology (Table I), 17 surgical specimens of glioblastomas, eight benign and two malignant meningiomas, 13 neurinomas, 12 medulloblastomas and four ependymomas. The tumour samples were collected directly from the operation room, and parts of the tumours were frozen in liquid nitrogen within 2 h of removal, and stored at −70°C until use. The remaining parts were fixed in 10% buffered formalin, and embedded in paraffin 'for conventional histopathological examination.

### RNA isolation and analyses

Total cellular RNA was isolated from the tumour samples and cell lines by the method of Chomczynski and Sacchi (1987). Northern blot analysis was done with a 1.5 kb cDNA probe encompassing both standard and variant CD44 sequences (Hofmann *et al.*, 1991). A spontaneously immortalised human keratinocyte line, HaCat (Hofmann *et al.*, 1991), was used as positive control for splicing variants of CD44 in the experiments.

To confirm the results from Northern analysis, RNA samples extracted from 22 brain metastases (BMTs) and some representative cases of each kind of primary brain tumours (PBTs) were subjected to RT-PCR. Random primers were used for first-strand cDNA synthesis and sense upstream (5'-CAGACCTGCCCAATGCCTTTGATGGGAC) and antisense downstream (5'-CAAAGCCAAGGCCAAGAGGGC-TGCC) primers were used for PCR amplification. These primers flank the highly variable extracellular region, where additional exons can be 'inserted' by alternative splicing (Tolg *et al.*, 1993). The reverse transcription reaction was performed at 37°C for 60 min, followed by incubation at 95°C for 5 min to inactivate the reverse transcriptase. PCR was done with the following parameters: 94°C for 5 min, then

**Table I** Detection of CD44 molecules in PBTs and BMTs

| | *CD44 expression* | |
|---|---|---|
| *Tumour* | *CD44s* | *CD44v* |
| Glioblastoma multiformes | 17 17 | 0 17 |
| Meningiomas[a] | 10 10 | 0 10 |
| Neurinomas | 13 13 | 0 13 |
| Medulloblastomas[b] | 12 12 | 0 12 |
| Ependymomas | 4 4 | 0 4 |
| Brain metastases | 21 22 | 22 26 |

[a]Eight benign, two malignant. [b]Non-metastatic.

Correspondence: H Li
Received 26 July 1994; revised 11 January 1995; accepted 8 February 1995

92°C for 40 s; 60°C for 40 s and 74°C for 90 s for 35 cycles, and finally, 75°C for 5 min. For Southern blot analysis, the PCR products were resolved by electrophoresis on 1.4% agarose gel, transferred to hybond $N^+$ nylon membrane (Amersham, UK) and hybridised with [$\alpha$-$^{32}$P]dATP-labelled probes specific for exons v8-v10. After washing and exposure, the filter was stripped by incubation in boiled distilled water for 1-2 h and rehybridised with a cDNA probe for exons v6/v7, providing identification of CD44s and CD44v (Hofmann *et al.*, 1991) respectively.

*Immunocytochemical stainings*

In order to show differential CD44 expression at protein level and rule out the possibility of post-translational modification including epitope masking, immunocytochemical staining was performed on frozen sections of 26 brain metastases and all primary brain tumours. A monoclonal antibody detecting both CD44s and CD44v (Oncogene Science, Uniondale, NY, USA) and a polyclonal antibody against CD44v (a generous gift from Karl-Heinz Heider, Germany) were used for screening the existence of CD44s and CD44v. For the CD44v-positive cases, CD44 molecules expressed in those tumours were further characterised with four monoclonal antibodies (MAbs) specifically recognising the epitopes encoded by variable spliced exons v5 (VFF-8), v6 (VFF-7), v7 (VFF-9 and -17) and v8-v10 (VFF-14) (Mackay *et al.*, 1994). Keratinocytes in a normal skin section were used as positive control for CD44v. The results were compared with that obtained from Northern and RT-PCR-Southern analyses.

### Results

Northern hybridisation revealed that 17 glioblastoma cases, ten meningiomas, 13 neurinomas, four ependymomas and 12 medulloblastomas express CD44 RNAs which were uniform in size, 5.0 kb, 2.2 kb and 1.8 kb, corresponding to CD44s transcripts (Li *et al.*, 1993). In contrast, heterogeneous hybridisation patterns were observed among 22 brain metastases: one was completely negative for CD44 expression, two were similar to that of PBTs, while 19 showed enlarged CD44 transcripts. In the last cases, six showed a similar hybridisation pattern to HaCat cells which express a CD44v RNA encompassing exons v3-v10. In comparison with HaCat, the remaining 13 cases gave smaller transcripts which hybridised with specific probes for exons v4/v5 and v6/v7 or v8-v10 only or both. The incidence of CD44 and CD44v detection in all of the tumour groups was 100% CD44s and 0% CD44v in 56 PBT samples, and 95% CD44s and 86% CD44v in 26 BMT samples (Table I). Patient numbers and the origins, histologies and CD44 expression patterns of brain metastases are listed in Table II.

In parallel with the data of Northern blot hybridisation, the results of RT-PCR (Figure 1) demonstrated that, when the filters were hybridised with the exon v8-v10-specific probe, only a proportion of BMTs were positive, in the form of three main bands ranging from 800 bp to 1.4 kb. When the same filter was rehybridised with the probe for exons v6/v7, 17 out of 19 cases with enlarged transcripts in Northern blot gave a positive hybridisation. No hybridisation of CD44v could be found among the PBT samples. When the same filter was rehybridised with the 1.5 kb cDNA probe of CD44, the samples from both BMTs and PBTs revealed a strong 409 bp band corresponding to the PCR product of CD44s.

Immunocytochemical staining showed that all PBTs studied were positive for CD44, but no positive staining for any form of CD44v could be observed. In contrast, the staining could be detected in 22 out of 26 brain metastases with either anti-CD44s/v antibody or anti-CD44v antibody. However, BM-712 and BM-678, which gave positive hybridisation for CD44s in Northern and RT-PCR, was negative in immunohistochemistry, indicating possible contamination of tumour RNA by normal tissues expressing CD44s, e.g. gliosis. CD44 molecules expressed by those 22 positive cases were further characterised with four MAbs specifically recognising the epitopes encoded by variably spliced exons v5, v6, v7 and v8-v10. It was found that 13 cases were positive for exons v5-v10 (Figure 2a), six were positive for exons v5-v7 but not for exons v8-v10 (Figure 2b) and three were positive for exons v8-v10 but almost negative for exons v5-v7 (Figure 2c). These results are in good agreement with those obtained in Northern and RT-PCR analyses (Table II).

**Table II** Distribution of CD44v isoforms in brain metastases with different origins

| Origin | Histology | (+) rates | Case no. (sex/age) | CD44v RNA | CD44v IC | (+) v-exons 3 | 4/5 | 6/7 | 8 | 9/10[a] |
|---|---|---|---|---|---|---|---|---|---|---|
| Lungs | Squamous | 4/4 | 312 (M, 57) | ND | + | + | + | + | + | + |
| | | | 492 (F, 68) | + | + | + | + | + | + | + |
| | | | 667 (M, 58) | + | + | + | + | + | + | + |
| | | | 799 (M, 62) | + | + | + | + | + | + | + |
| | Adenocarcinoma | 3/4 | 375 (M, 64) | + | | + | + | | | |
| | | | 643 (M, 54) | + | | | | | + | + |
| | | | 671 (M, 52) | + | + | + | + | + | + | + |
| | | | 712 (M, 53) | − | − | | (−) | | | |
| | SCLC[b] | 0/2 | 678 (M, 55) | − | − | | (−) | | | |
| | | | 681 (M, 51) | − | − | | (−) | | | |
| | LCLC[b] | 1/1 | 544 (M, 44) | + | + | | + | + | | |
| Breast | Adenocarcinoma | 6/6 | 527 (F, 65) | ND | + | | + | + | + | + |
| | | | 663 (F, 58) | + | + | | + | + | + | + |
| | | | 710 (F, 56) | + | + | | + | + | | |
| | | | 725 (F, 58) | + | + | | + | + | | |
| | | | 778 (F, 70) | + | + | | + | + | + | + |
| | | | 836 (F, 51) | + | + | | + | + | + | + |
| Testis | Adenocarcinoma | 2/2 | 687 (M, 51) | + | + | | | | + | + |
| | | | 748 (M, 50) | ND | + | | | | + | + |
| Cervix | Squamous | 1/1 | 345 (F, 56) | + | + | + | + | + | + | + |
| Tonsil | Squamous | 1/1 | 532 (M, 63) | + | + | + | + | + | + | + |
| Colorectum | Adenocarcinoma | 2/2 | 395 (M, 60) | + | + | | + | + | | |
| | | | 863 (F, 53) | + | + | | + | + | | |
| Histiocyte | Histiocytoma | 1/1 | 733 (F, 58) | + | + | | + | + | + | + |
| Skin | Melanoma | 1/1 | 463 (M, 66) | + | + | | + | + | + | + |
| Kidney | Adenocarcinoma | 0/1 | 318 (M, 61) | ND | − | | | | (−) | |
| Positive rates | | 22/26 (84%) | | 19/22 | 22/26 | | | | | |

[a]v3, domain I; v4/v5, II; v6/v7, III; v8, IV; v9/v10, V. [b]SCLC, small-cell lung cancer; LCLC, large-cell lung cancer.

**Figure 1** Representation of differential CD44 expression patterns in human brain metastases (BMs) and primary brain tumours (PBTs). HaCat, the positive control for CD44 v3–v10. BMs: 1, a squamous cell carcinoma of the tonsil (no. 532); 2, an adenocarcinoma of the lung (no. 375); 3, an adenocarcinoma of the breast (no. 710); 4, a squamous cell carcinoma of the lung (no. 667) and 5, a small-cell lung cancer (no. 681). PBTs: G, a case of glioblastoma; M, meningioma; N, neurinoma; Md, medulloblastoma; and E, ependymoma.

### Discussion

The current study provides further evidence for the rarity of CD44v expression in primary brain tumours, suggesting that lack of CD44 variants may be of biological significance in the low propensity of these tumours to metastasise. This study does not exclude the possibility that CD44s, as a hyaluronate-binding adhesion molecule, may play a role in local tumour invasion and spreading, since hyaluronate is a major component of brain extracellular matrix (Asher and Bignami, 1992). However, the variable invasive behaviour of PBTs suggests that this may be the case only after additional genetic event(s) have occurred, increasing the malignancy of the cells. Additionally, our data imply that CD44v may not be necessary for brain tumour invasion because of its absence in highly invasive glioblastomas.

According to the literature, CD44v is expressed constitutively in squamous cells of normal bronchial, cervical and tonsil mucosa, but not in normal epithelial cells in the colon, lung and breast (Heider *et al.*, 1993*a*; Mackay *et al.*, 1994), demonstrating a cell type-specific expression of CD44v in normal epithelial cells. In contrast, CD44v becomes detectable in metastatic tumours including brain metastases formed by adenocarcinomas of the breast (6/6), the lung (3/4) and digestive tract (2/2), supporting the idea that CD44v expression is acquired during progression of human adenocarcinomas (Heider *et al.*, 1993*b*). Furthermore, our results demonstrate that, as well as keratinocytes grown *in vivo* and *in vitro*, all brain metastases of squamous cell origin expressed full-length CD44v, ruling out possible instability or down-regulation of CD44v expression after malignant transformation of squamous epithelial cells (Salmi *et al.*, 1993).

As shown in Table II, CD44v is distributed in 84% of various kinds of brain metastases with different morphologies and its expression pattern is heterogeneous: squamous cell carcinomas, irrespective of origin from the lungs, tonsil or cervix, express mainly CD44v containing exons v3–v10, while adenocarcinomas express multiple isoforms with exons v4–v7, v8–v10 or v3–v10. Since exons v4–v7 are found to exist in most of the tumours studied, it is possible that these

**Figure 2** Immunocytochemical stainings with the monoclonal antibodies against CD44v exon 5, VFF-8 (left) and exons 8–10, VFF-14 (right). (**a**) Brain metastasis originating from a squamous cell carcinoma of the cervix (BM-345). (**b**) Brain metastasis formed by adenocarcinoma of the lungs (BM-643). (**c**) A brain metastasis formed by a large cell lung cancer (BM-544). (**d**) Normal skin as positive control for both v5 and v8–v10.

exons may be the structural and even functional core part of CD44v. Absence of CD44s and CD44v expression in the remaining 16% of brain metastases formed by small-cell lung cancers, adenocarcinomas of the lungs and kidney implies the existence of CD44v-independent metastatic pathways which may be mediated by other genetic alterations.

In summary, our data present direct evidence for general distribution of CD44v in various kinds of human tumours metastasising to the brain and show that expression of CD44v is an extremely rare event in five types of PBTs, indicating that CD44v may be one of the important elements for tumour cells to metastasise.

**Abbreviations**: CD44s, standard form of CD44; CD44v, splicing variants of CD44; RT-PCR, reverse transcription-polymerase chain reaction.

**Acknowledgements**
We thank Drs Karl-Heinz Heider and Petra Skroch-Angel for providing polyclonal antibody against CD44v and Dr E Patzelt for providing monoclonal antibodies against variant exons of CD44. We would also like to thank Dr Geoffrey J Pilkington for his critical reading of the manuscript. This work was supported in part by the Swiss National Science Foundation and by the National Natural Science Foundation (39470767) of the People's Republic of China.

### References

ARCH R, WIRTH K, HOFMANN M, PONTA H, MATSKU S, HERRLICH P AND ZOLLER M. (1992). Participation in normal immune responses of a metastasis-inducing splice variant of CD44. *Science*, **257**, 682-685.

ASHER R AND BIGNAMI A. (1992). Hyaluronate binding and CD44 expression in human glioblastoma cells and astrocytes. *Exp. Cell Res.*, **203**, 80-90.

CHOMCZYNSKI P AND SACCHI N. (1987). Single-step method of RNA isolation by acid guanidinium thiocyanate-phenol-chloroform extraction. *Anal. Biochem.*, **162**, 156-159.

GUNTHERT U, HOFMANN M, RUDY W, REBER S, ZOLLER M, HAUßMANN I, MATZKU S, WENZEL A, PONTA H AND HERRLICH P. (1991). A new variant of glycoprotein CD44 confers metastatic potential to rat carcinoma cells. *Cell*, **65**, 343-348.

HEIDER K-H, HOFMANN M, HORS E, VAN DEN BERG F, HERRLICH P AND PALS ST. (1993*a*). A human homologous of the rat metastasis-associated variant of CD44 is expressed in colorectal carcinomas and adenomatous polyps. *J. Cell Biol.*, **120**, 227-233.

HEIDER K-H, DAMMARICH J, SKROCH-ANGEL P, MULLER-HERMELINK H-K, VOLLMERS HP, HERRLICH P AND PONTA H. (1993*b*). Differential expression of CD44 splice variants in intestinal- and diffuse-type human gastric carcinomas and normal gastric mucosa. *Cancer Res.*, **53**, 4197-4203.

HOFMANN M, RUDY W, ZOLLER M, TOLG C, PONTA H, HERRLICH P AND GUNTHERT U. (1991). CD44 splice variants confer metastatic behaviour in rat: homologous sequences are expressed in human tumour cell lines. *Cancer Res.*, **51**, 5292-5297.

LI H, HAMOU M-F, DE TRIBOLET N, HOFMANN M, DISERINSE A-C AND VAN MEIR EG. (1993). Variant CD44 adhesion molecules are expressed in brain metastases but not in glioblastomas. *Cancer Res.*, **53**, 5345-5349.

MACKAY CR, TERPE H-J, ATAUDER R, MARSTON WJ, STARK H AND GUNTHERT U. (1994). Expression and modulation of CD44 variant isoforms in humans. *J. Cell Biol.*, **124**, 71-82.

MATSUMURA Y AND TARIN D. (1992). Significance of CD44 gene products for cancer diagnosis and disease evaluation. *Lancet*, **340**, 1053-1058.

MAYER B, JAUCH KW, GUNTHERT U, FIGDOR CG, SCHILDBERG FW, FUNKE I AND JOHNSON J. (1993). *De-novo* expression of CD44 and survival in gastric cancer. *Lancet*, **342**, 1019-1022.

SALMI M, GRON-VIRTA K, SOINTU P, GRENMAN R, KALIMO H AND JALKANEN S. (1993). Regulated expression of exon v6 containing isoforms of CD44 in man: down regulation during malignant transformation of tumours of squamocellular origin. *J. Cell Biol.*, **122**, 431-442.

TOLG C, HOFMANN M, HERRLICH P AND PONTA H. (1993). Splicing of choice from ten variant exons establishes CD44 variability. *Nucleic Acids Res.*, **21**, 1225-1229.

WIELENGA VJM, HEIDER K-H, OFFERHAUS GJA, ADOLF GR, VAN DEN BERG FM, PONTA H, HERRLICH P AND PALS ST. (1993). Expression of CD44 variant proteins in human colorectal cancer is related to tumour progression. *Cancer Res.*, **53**, 1754-1756.